# Zoledronic acid for chronic kidney disease–associated osteoporosis

**DOI:** 10.1007/s11657-026-01680-2

**Published:** 2026-02-25

**Authors:** Sukanya Sakthivel, Krista Dybtved Kjærgaard, Per Ivarsen, Bente Langdahl, Hanne Skou Jørgensen

**Affiliations:** 1 Department of Renal Medicine, Aarhus University Hospital, Aarhus, Denmark; 2 Department of Clinical Medicine, Aarhus University, Aarhus, Denmark; 3 Department of Endocrinology and Internal Medicine, Aarhus University Hospital, Aarhus, Denmark

**Keywords:** Bone density, Bone fracture, Chronic kidney disease–mineral and bone disorder, Chronic renal insufficiencies, Dialysis, Osteoporosis, Zoledronic acid

## Abstract

**Background:**

Patients with chronic kidney disease have a high risk of fracture, but evidence-based treatment protocols for osteoporosis are lacking. Bisphosphonates are commonly used anti-resorptive drugs, but due to renal excretion, patients with chronic kidney disease have not been included in clinical trials.

**Case presentation:**

We present a case of severe bone fragility in a patient on long-term hemodialysis therapy where zoledronate was well-tolerated and provided clinical improvement.

**Conclusion:**

This case highlights the potential role of bisphosphonates in chronic kidney disease–associated osteoporosis and the need for trials to establish the safety and efficacy of anti-resorptive drugs in this patient population.

## Introduction

Chronic kidney disease (CKD) is closely linked to osteoporosis and bone fragility, with patients on dialysis facing a markedly elevated fracture risk [1]. The pathophysiology of CKD-associated osteoporosis is multifactorial, involving disturbances in mineral metabolism, chronic inflammation, drug use, and comorbidities [2]. Despite the burden of fractures, there are no established treatment algorithms for CKD-associated osteoporosis, resulting in a large treatment gap [3]. Anti-resorptive therapies are the cornerstone of osteoporosis management [4], yet their use in CKD is challenging. Denosumab, while not renally excreted, carries a high risk of potentially life-threatening hypocalcemia, particularly in kidney failure [5]. Bisphosphonates, traditionally avoided due to renal clearance and nephrotoxicity, may yet be suitable for patients with CKD and little or no residual kidney function. Zoledronate (ZOL), administered intravenously, offers practical advantages for patients receiving dialysis and may provide a safe anti-resorptive option. This report describes a patient with kidney failure receiving dialysis who suffered multiple vertebral fractures, reflecting severe bone fragility. Following multidisciplinary evaluation, treatment with reduced-dose ZOL was initiated, which led to bone densitometric and clinical improvement.

## Case report

**Fig. 1:**
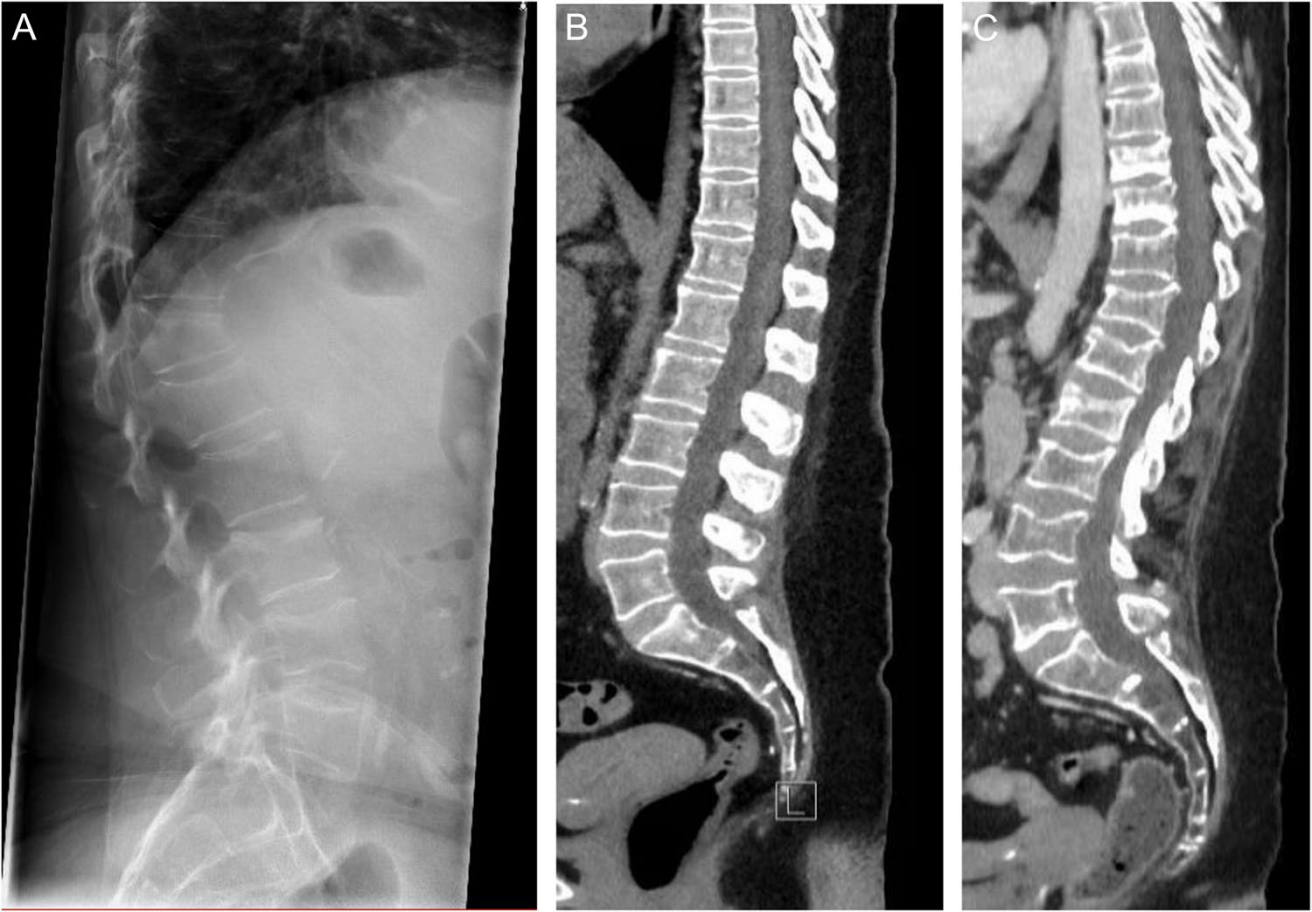
Imaging of vertebral fractures. **A** Plain X-ray of the lumbar spine shortly after fourth vertebral fracture. **B** Sagittal reconstruction of spinal computed tomography images performed 2 years prior to the first fracture. **C** Sagittal reconstruction of spinal computed tomography images 1 year after the first fracture

A 56-year-old woman with kidney failure due to polycystic kidney disease, who had received in-center hemodialysis for > 10 years, presented with sudden severe back pain. Imaging revealed fractures of L3 and L4. Within the following month, she developed an additional fracture of Th12, and 5 months later a fourth fracture of L2 (Fig. 1). After initial management with analgesics and physiotherapy, she was referred to a multidisciplinary nephrology–endocrinology team for consideration of anti-osteoporosis therapy.

Her medical history was notable for multiple comorbidities and complications, including subtotal parathyroidectomy for severe secondary hyperparathyroidism (9 years prior), calciphylaxis (6 years prior), ischemic heart disease (2 years prior), breast cancer treated surgically (< 1 year prior), anti-phospholipid syndrome requiring anticoagulation, and intermittent corticosteroid therapy for arthritis. She had no residual kidney function and had not been eligible for transplantation until recently, when a significant weight loss (> 10 kg with the use of a GLP-analog) had improved her candidacy.

**Fig. 2:**
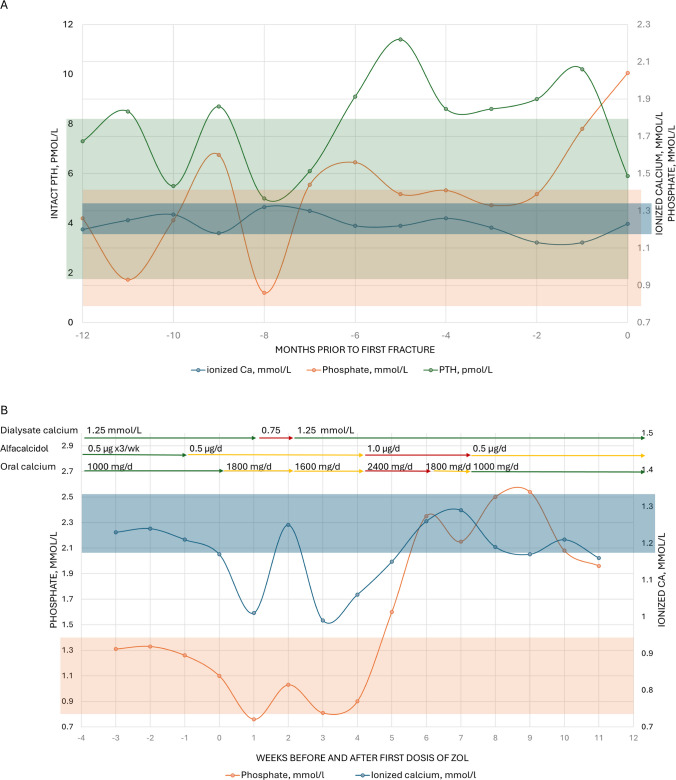

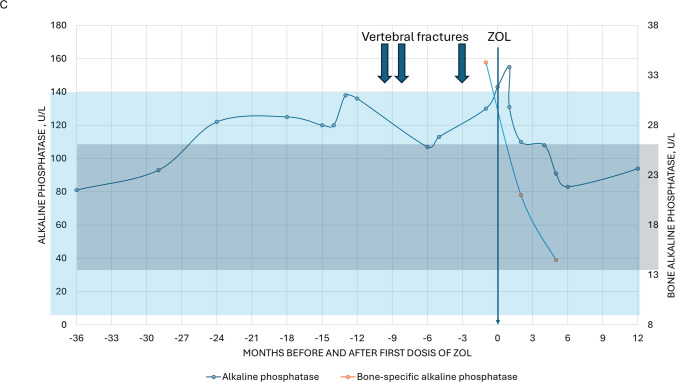
**A** Trends 1 year prior to the first vertebral fracture, PTH (left axis, green), phosphate (right axis, orange), and ionized calcium (right axis, blue). **B** Change in phosphate (left axis, orange), ionized calcium (right axis, blue), and medical therapy before and after initial dose of ZOL. **C** Change in total (left axis, light blue) and bone-specific (right axis, dark blue) alkaline phosphatase before and after initial dose of ZOL

Bone imaging demonstrated multiple vertebral fractures (Fig. 1), and dual-energy X-ray absorptiometry (DXA) showed osteoporosis at both the spine (T-score −3.2) and hip (T-score −3.5). Laboratory results revealed normal to slightly elevated parathyroid hormone, with calcium, phosphorus, and vitamin D in the target range (Fig. 2A). Serum alkaline phosphatase (ALP; normal range 40 to 120 U/L) gradually increased over time to 130–140 U/L just prior to the first fracture, indicating high bone turnover. Hematological parameters were normal. C-reactive protein was consistently elevated in the range of 10–50 mg/L. Gonadal function was consistent with post-menopausal status. The multidisciplinary team concluded that bone fragility was multifactorial, related to CKD, previous hyperparathyroidism, menopause, weight loss, chronic inflammation, and corticosteroid exposure. Anabolic therapy was discussed but considered contraindicated due to cardiovascular disease and previous malignancy. Denosumab was deemed high-risk due to the potential for severe hypocalcemia. Instead, reduced-dose intravenous ZOL (2.5 mg annually, over 30 min) was recommended. The patient was informed and consented to the off-label use of ZOL.

Following the first infusion of ZOL, the patient developed a hungry bone response with transient hypocalcemia and hypophosphatemia (Fig. 2B), which was managed with increased dialysate calcium and alfacalcidol, oral calcium supplementation, and dietary advice regarding phosphate intake. The patient did not experience any clinical symptoms of hypocalcemia. Calcium and phosphorus normalized within 4 weeks, and bone and total ALP decreased to normal range over 6 months (Fig. 2C; bone ALP normal range 8.3 to 29.4 μg/L). At 1-year follow-up, ALP was in the normal range, and DXA showed BMD increases of 5.4% at the left and 1.1% at the right total hip, with no change in lumbar spine BMD, which, however, was considered unreliable due to the multiple vertebral fractures. No new episodes of back pain had occurred, and no new vertebral fractures were identified on a CT scan. A mild recurrence of hypocalcemia followed the second infusion of ZOL but was easily managed with oral medication.

## Discussion

This case illustrates the complex, multifactorial nature of osteoporosis in kidney failure and the practical considerations that guide therapy in patients receiving dialysis. This patient had long-standing CKD with > 10 years of hemodialysis and experienced multiple vertebral fractures over a short time period. The bone phenotype likely reflected cumulative skeletal injury from prior severe secondary hyperparathyroidism, compounded by corticosteroid exposure, early post-menopausal status, and ongoing chronic inflammation. The recent weight loss under GLP1-analog therapy may also have contributed [6]. Mineral metabolism parameters were within target, though PTH was relatively low for kidney failure [7]. Serial measurements showed that ALP had been moderately elevated in the year preceding the first fracture, suggesting high bone turnover.

When treating CKD-associated osteoporosis, correction of mineral metabolism abnormalities (calcium, phosphate, vitamin D) and other reversible contributors (glucocorticoid therapy) is crucial before initiating bone-targeting drugs. Both anti-resorptive and bone anabolic drugs may improve BMD in CKD, but the evidence base is limited and relies largely on observational data [8]. Choice of therapy must balance the benefit of fracture risk reduction against the risks pertinent to kidney failure such as hypocalcemia, nephrotoxicity, and adynamic bone. In this case, bone anabolic therapy was considered because of rapid, severe bone fragility and comparatively low PTH; however, romosozumab and teriparatide were considered contraindicated due to established cardiovascular disease and previous malignancy, respectively.

Among the anti-resorptives, denosumab is pharmacokinetically attractive in CKD due to non-renal clearance but clinically constrained by a high and recurrent risk of severe hypocalcemia, particularly in kidney failure [9], necessitating vigilant monitoring after every dose. Further, denosumab cannot be discontinued without risk of rebound bone resorption upon discontinuation [10]. By contrast, the bisphosphonates offer a substantially lower incidence of hypocalcemia [9] with the option to stop therapy without risk of rebound. Further, ZOL can be given once yearly following a dialysis session, thus avoiding additional pill burden. Although concerns have been raised regarding nephrotoxicity, this risk has mainly been found with high cumulative dosing used in oncology [11, 12], while real-world osteoporosis regimens show low rates of kidney injury, that is often reversible [13]. In anuric or near-anuric patients receiving dialysis, the clinical relevance of nephrotoxicity is attenuated, though residual kidney function—when present—still warrants caution. Available data suggests dialyzability of intravenously administered alendronate [14], arguing against excessive tissue accumulation. Given the paucity of randomized data in kidney failure, a cautious strategy with reduced-dose and close biomarker surveillance seems reasonable.

We selected ZOL 2.5 mg annually with quarterly monitoring of bone turnover biomarkers and DXA at 12 months to evaluate response. Shortly after the first infusion, the patient developed a *hungry bone* response—parallel declines in calcium and phosphate—managed with higher dialysate calcium, alfacalcidol, oral calcium, and dietary measures. Electrolytes normalized within 4 weeks, and dialysate calcium could be de-escalated. Total and bone-specific ALP decreased into the normal range by 6 months. At 12 months, BMD increased substantially at the lumbar spine—less so at the hip. Most importantly, no new clinical fractures were observed. These findings suggest effective suppression of bone resorption with clinical benefit for the patient.

Concerns specific to CKD deserve emphasis. First, adynamic bone remains a theoretical risk with anti-resorptives; however, clear signals of harm with very low turnover are lacking, and the rare occurrence of atypical femoral fractures with long-term therapy does not appear amplified by CKD [15]. Second, while low turnover has been linked to vascular calcification progression in CKD [16], bisphosphonate use itself has not shown a pro-calcific signal and may be neutral or even beneficial for cardiovascular outcomes [17]. Third, anti-resorptives are contraindicated in osteomalacia [18]; however, overt osteomalacia appears uncommon in contemporary CKD cohorts [19, 20], and careful biochemical review and bone histomorphometry in atypical cases can mitigate this risk. Finally, the pharmacokinetics of ZOL in patients with kidney failure has not been investigated. A previous study demonstrated that the removal of intravenous alendronate, a bisphosphonate of similar molecular weight to ZOL, was 51% during a single hemodialysis session [14], which is nearly equal to renal clearance in individuals with normal kidney function, which argues against the risk of excessive accumulation of bisphosphonates in patients with kidney failure receiving hemodialysis. Further studies should be undertaken to investigate the pharmacokinetics of ZOL in relation to dialysis therapy, specifically. These considerations reinforce the importance of individualized therapy, meticulous biochemical optimization, and ongoing monitoring of patients receiving dialysis and undergoing anti-osteoporotic treatment.

In summary, for this high-risk patient with kidney failure and clinical bone fragility, reduced-dose ZOL provided a pragmatic balance: presumed lower hypocalcemia risk than denosumab, convenient once-yearly dosing that could be administered in the dialysis unit, improvements in BMD, and clinical stability after 12 months. While single-case data cannot define standards of care, this experience supports further systematic study of the safety and efficacy of bisphosphonates in CKD-associated osteoporosis, with particular interest in dose-finding, safety for residual kidney function, and predictors of response.

Until randomized evidence emerges, a cautious ZOL protocol with pre-treatment biochemical optimization, slow infusion, close electrolyte monitoring, and biomarker-guided follow-up appears to be a feasible option for selected patients with kidney failure and bone fragility.

## Conclusion

Fracture risk is high in patients with kidney failure, but evidence-based treatment guidelines are lacking. Denosumab, though not contraindicated, carries a significant risk of severe hypocalcemia, particularly in the setting of dialysis. ZOL, an intravenous bisphosphonate, may be a pragmatic choice, offering a more beneficial safety profile. This case demonstrates that ZOL was relatively well tolerated and effective in severe bone fragility in a patient with kidney failure receiving dialysis therapy. Further studies are needed to clarify the safety and efficacy of bisphosphonates in CKD-associated osteoporosis.

## Data Availability

The dataset is available from the corresponding author upon reasonable request.

## Declarations

Written patient consent was obtained prior to publication of this manuscript.
